# Physicochemical Factors That Favor Conjugation of an Antibiotic Resistant Plasmid in Non-growing Bacterial Cultures in the Absence and Presence of Antibiotics

**DOI:** 10.3389/fmicb.2018.02122

**Published:** 2018-09-11

**Authors:** Brendan Headd, Scott A. Bradford

**Affiliations:** U.S. Salinity Lab, United States Department of Agriculture (USDA), Agricultural Research Service (ARS), Riverside, CA, United States

**Keywords:** conjugation, horizontal gene transfer, antibiotic resistance, salinity, β-lactam antibiotics, non-growing bacteria

## Abstract

Horizontal gene transfer (HGT) of antibiotic resistance genes has received increased scrutiny from the scientific community in recent years owing to the public health threat associated with antibiotic resistant bacteria. Most studies have examined HGT in growing cultures. We examined conjugation in growing and non-growing cultures of *E. coli* using a conjugative multi antibiotic and metal resistant plasmid to determine physiochemical parameters that favor horizontal gene transfer. The conjugation frequency in growing and non-growing cultures was generally greater under shaken than non-shaken conditions, presumably due to increased frequency of cell collisions. Non-growing cultures in 9.1 mM NaCl had a similar conjugation frequency to that of growing cultures in Luria-Bertaini broth, whereas those in 1 mM or 90.1 mM NaCl were much lower. This salinity effect on conjugation was attributed to differences in cell-cell interactions and conformational changes in cell surface macromolecules. In the presence of antibiotics, the conjugation frequencies of growing cultures did not increase, but in non-growing cultures of 9.1 mM NaCl supplemented with Cefotaxime the conjugation frequency was as much as nine times greater than that of growing cultures. The mechanism responsible for the increased conjugation in non-growing bacteria was attributed to the likely lack of penicillin-binding protein 3 (the target of Cefotaxime), in non-growing cells that enabled Cefotaxime to interact with the plasmid and induce conjugation. Our results suggests that more attention may be owed to HGT in non-growing bacteria as most bacteria in the environment are likely not growing and the proposed mechanism for increased conjugation may not be unique to the bacteria/plasmid system we studied.

## Introduction

Antibiotic resistance has become a serious public health issue (de Kraker et al., 2011; Smith and Coast, 2013) and the spread of antibiotic resistant genes (ARG) via horizontal gene transfer (HGT) is a topic that has received increased scrutiny by the scientific community in recent years. There is a growing concern that the presence of antibiotics in the environment can act as a stressor to induce HGT of ARG. Varying concentrations of antibiotics have been shown to be prevalent in the environment (Ohlsen et al., 2003; Karthikeyan and Meyer, 2006; Sarmah et al., 2006; Martínez-Carballo et al., 2007; Guerra et al., 2014) and numerous studies have correlated the use (and misuse) of antibiotics in agriculture and medicine with an increase in antibiotic resistant bacteria (Khachatourians, 1998; Smith et al., 2002; Cabello, 2006; Centers for Disease Control Prevention, 2013; Ventola, 2015). Antibiotics have also been shown to induce HGT of ARG in both field and laboratory based studies (Barr et al., 1986; Hinnebusch et al., 2002; Licht et al., 2003; Ohlsen et al., 2003; Maiques et al., 2006; Pontiroli et al., 2009; Stecher et al., 2012; Zhang et al., 2013; Kim et al., 2014; Schuurmans et al., 2014; Jutkina et al., 2018).

Despite the abundant number of studies examining HGT of ARG via conjugation, the results (and methods) from these studies vary greatly. Some studies have reported that subinhibitory concentrations of antibiotics are capable of inducing HGT (Barr et al., 1986; Licht et al., 2003; Maiques et al., 2006; Kim et al., 2014; Jutkina et al., 2016, 2018), while other studies report they are not (Ohlsen et al., 2003; Lopatkin et al., 2016) and still other studies report that antibiotic concentrations above the minimum inhibitory concentration are necessary to promote HGT (Schuurmans et al., 2014; Moller et al., 2017). Rarely are the same methods used by investigators to assess conjugation. For example, some studies have chosen to shake cultures (Zhang et al., 2013; Händel et al., 2015), while other studies have chosen not to shake cultures (Hayashi et al., 1988; Fernandez-Astorga et al., 1992; Muela et al., 1994; Beuls et al., 2012; Sandegren et al., 2012; Lopatkin et al., 2016). Some studies have examined conjugation in liquid cultures (Barr et al., 1986; Fernandez-Astorga et al., 1992; Sandegren et al., 2012; Zhang et al., 2013; Schuurmans et al., 2014; Händel et al., 2015; Lopatkin et al., 2016), while others have examined conjugation using the filter plate method (Kim et al., 2014; Jutkina et al., 2016, 2018; Moller et al., 2017). Many studies have examined conjugation at 37°C (Khalil and Gealt, 1987; Hayashi et al., 1988; Fernandez-Astorga et al., 1992; Sandegren et al., 2012; Zhang et al., 2013; Moller et al., 2017), while many other investigators have utilized other temperatures for their experiments (Fernandez-Astorga et al., 1992; Muela et al., 1994; Beuls et al., 2012; Jutkina et al., 2016; Lopatkin et al., 2016; p. 74; Jutkina et al., 2018).

One area of methodological agreement appears to be the use of growing bacterial cultures in conjugation experiments. There is good evidence that conjugation increases with bacterial growth (Muela et al., 1994; Schuurmans et al., 2014; Händel et al., 2015; Lopatkin et al., 2016) and the vast majority of conjugation studies are conducted on growing bacterial populations or populations exposed to growth media during conjugation (e.g., Barr et al., 1986; Hayashi et al., 1988; Fernandez-Astorga et al., 1992; Beaber et al., 2004; Beuls et al., 2012; Sandegren et al., 2012; Zhang et al., 2013; Kim et al., 2014; Schuurmans et al., 2014; Händel et al., 2015; Jutkina et al., 2016, 2018; Lopatkin et al., 2016; Matsumoto et al., 2016; Moller et al., 2017). However, just because conjugation has been shown to increase with bacterial growth does not mean that it does not occur in non-growing bacteria. To our knowledge, there has been relatively little work on conjugation with non-growing bacteria, this is likely in part due to the perception that bacteria must be in an environment suitable for bacterial growth for conjugation to occur (van Elsas and Bailey, 2002; Aminov, 2011). Studies of conjugation in nutrient depleted natural and artificially created environments have been conducted (Grabow et al., 1975; Gowland and Slater, 1984; O'Morchoe et al., 1988; Jones et al., 1991; Sandt and Herson, 1991; Goodman et al., 1993; Dahlberg et al., 1998; Coombs and Barkay, 2004) and have demonstrated that conjugation can occur under these (perceived) less than favorable conditions. However, these studies tended to only examine conjugation at the physiochemical conditions that existed in the natural environment (or artificially created environment designed to mimic the natural environment) to determine if conjugation was occurring, not determine the optimal conditions for it to occur. It has been suggested that the factors promoting conjugation in growing bacteria may be different than those in non-growing bacteria (Goodman et al., 1994), thus an examination of the physiochemical parameters in which conjugation occurs in non-growing bacteria is warranted.

We examined conjugation in growing and non-growing cultures of *E. coli* using a conjugative multi antibiotic and metal resistant plasmid to determine the physiochemical conditions that most favor the transfer of the plasmid to recipient bacteria. We found that shaken cultures generally produced much more conjugation than non-growing cultures. The conjugation frequency of non-growing cultures suspended in 9.1 mM NaCl was equal to that of growing cultures in Luria-Bertaini (LB) broth at 37°C. In the presence of 25 μg/ml of Cefotaxime, the conjugation frequency exceeded the conjugation frequency of growing cultures in LB by as much as nine times. Only β-lactam antibiotics increased conjugation frequencies and subinhibitory concentrations of antibiotics generally resulted in either similar or less conjugation than non-antibiotic control cultures.

## Materials and methods

### Bacterial strains

*E. coli* K12 ER1793 is a streptomycin resistant and restriction enzyme deficient strain obtained from New England Biolabs (Ipswich, MA) and was chromosomally modified to contain either a blue (mTagBFP2) or yellow (SYFP2) fluorescent gene and a chloramphenicol resistance gene as described below in the strain modification section.

*E. coli* DA28100 and *E. coli* DA28102 were derived from wild-type *E. coli* MG1655 strains and provided by Dr. Erik Gullberg at Uppsala University, Sweden. *E. coli* strain DA28100 was modified (as described in Gullberg et al., 2014) to possess a chromosomal SYFP2 gene and a chloramphenicol resistance gene. *E. coli* strain DA28102 was modified to possess a chromosomal mTagBFP2 gene and a chloramphenicol resistance gene.

pUUH239.2 was provided by Dr. Linus Sandegren at Uppsala University, Sweden in an *E.coli* K-12 strain (DA14833). The plasmid was originally isolated from a *Klebsiella pneumoniae* strain associated with a nosocomial outbreak in Uppsala, Sweden. pUUH239.2 is a 220,884 bp conjugative plasmid that encodes resistance to multiple antibiotics (e.g., B-lactams, tetracycline, kanamycin), a variety of heavy metals (e.g., copper, silver) and organic biocides (Sandegren et al., 2012). The plasmid has been fully sequenced and characterized with respect to its ability to be transferred via conjugation, associated fitness costs, and antibiotic and metal concentrations necessary to select for the plasmid (Sandegren et al., 2012; Gullberg et al., 2014).

### Antibiotics

Minimum Inhibitory Concentration (MIC) assays of strains used in conjugation experiments were carried out in Mueller Hinton broth (Fisher Scientific, Waltham, MA, USA) using the method similar to European Committee for Antimicrobial Susceptibility Testing (EUCAST) of the European Society of Clinical Microbiology, and Infectious Diseases (ESCMID) (2003) by diluting antibiotics 1:10 from 100 μg to 0.01 μg/ml (Supplementary Table 1). The goal of the MIC assays was not to determine the actual MIC, but to establish selective and subinhibitory concentrations of antibiotics for use in conjugation experiments. Subinhibitory concentrations used in conjugation experiments were 1/10th of the determined MIC for each antibiotic. The following antibiotics were used to maintain strains and/or used in conjugation experiments: Ampicillin Sodium Salt (AMP) (Fisher Scientific, Waltham, MA, USA); Cefotaxime Sodium Salt (CFX) (ACROS Organics, Thermo-Fisher Scientific, Waltham, MA, USA); Kanamycin Monosulfate (KAN) (Fisher Scientific, Waltham, MA, USA); Gentamicin Sulfate (GEN) (Sigma-Adrich, St Louis, MO, USA); Tetracycline HCl (TET) (RPI, Mount Prospect, IL, USA); and Chloramphenicol (CHL) (Fisher Scientific, Waltham, MA, USA).

### Strain modification

Creation of *E. coli* K12 ER1793 galk::mTagBFP2-FRT and E. coli K12 ER1793 galk::SYFP2-Cat was carried out as described by Gullberg et al. (2014). Briefly, *E. coli* K12 ER1793 was transformed with pkD46 (Coli Genetics Stock Center, Yale University), a 6,329 bp plasmid containing the genes necessary for lambda red recombination. The recombination was carried out to insert a fluorescent gene (mTagBFP2 or SYFP2) and a chloramphenicol resistance gene flanked by Flp recombination (FRT) sites into the *galK* (galactokinase) gene on the chromosome of *E. coli* K12 ER1793. The insertion site comprises a region spanning the start codon of *galK* to 116 bp upstream of the 3' end of the gene. Chromosomal DNA of *E. coli* DA28100 and *E. coli* DA28102 was extracted using the QIamp Kit (Qiagen, Germantown, MD) and amplified with pcr primers (pELgalKEcoF and ColiGalKYFPLinR from Gullberg et al. (2014)) to generate an approximately 2050 bp fragment containing a chloramphenicol resistance gene and either a mTagBFP2 gene or a SYFP2 gene. The fragments were extracted from a gel using the Wizard SV Gel and PCR Clean-up system (Promega Corporation, Madison, WI). Approximately 50 μg of pcr product was transformed into *E. coli* K12 ER1793//pKD46 via electroporation (2.0 kV, 200 ohms, and 25 μF for ~5 mS) and immediately incubated with 1 ml of LB broth for ~2 h at 37°C and ~200 rpm before plating 100 μl onto LB agar supplemented with CHL (20 μg/ml) overnight at 37°C. Successful recombinants were restreaked onto LB agar plates supplemented with CHL (20 μg/ml) and incubated overnight at 42°C to remove the pkD46 plasmid. The resulting recombination was then moved into the wild-type *E. coli* K12 ER1793 chromosome via P1 transduction using the P1vir phage (Coli Genetics Stock Center, Yale University) to generate *E. coli* K12 ER1793 galk::mTagBFP2-cat and E. coli K12 ER1792 galk::SYFP2-cat (herein referred to as ER1793_SYFP2-Cat). *E. coli* K12 ER1793 galk::mTagBFP2-cat was then transformed with pCP20 (Coli Genetics Stock Center, Yale University), a 9,497 bp plasmid containing the yeast Flp recombinase gene. The chloramphenicol resistance gene was then removed from the strain using the FRT sites to generate *E. coli* K12 ER1793 galk::mTagBFP2-FRT. pCP20 was removed by incubating the strain overnight at 42°C. The area of the modified chromosomes of both strains was then sequenced to confirm successful modification of the chromosomes. *E. coli* K12 ER1793 galk::mTagBFP2-FRT was then transformed with pUUH239.2 via electroporation (2.0 kV, 200 ohms, and 25 uF for ~5 mS) to generate E. coli K12 ER1793 galk::mTagBFP2FRT//pUUH239.2 (herein referred to as ER1793_mTag/pUUH239.2) which was grown and maintained on LB agar supplemented with CFX (25 μg/ml).

### Conjugation experiments

ER1793_mTag/pUUH239.2 and ER1793_SYFP2-Cat were grown overnight in 10 ml of LB broth (Fisher Scientific, Waltham, MA, USA) supplemented with 150 μg/ml CFX and 20 μg/ml CHL, respectively, at 37°C and ~200 rpm. One ml of each overnight culture was then inoculated into 34 ml of LB broth (supplemented with 25 μg/ml CFX for ER1793 mTag/pUUH239.2) and grown at 37°C for approximately 4.5 h to an OD600 of between 0.400 and 0.600. Cultures were then centrifuged at 5,100 rpm at 22°C for 15 min and pellets resuspended in 35 ml of 1 mM NaCl. The OD600 was then adjusted using 1 mM NaCl to ensure that both cultures were within an OD600 of 0.01 of one another. 500 μl of each culture was then inoculated into 9 ml of LB, 1, 10, and 100 mM NaCl (to generate 1, 9.1, and 90.1 mM NaCl solutions) with and without antibiotics and incubated at the experimental temperature for 2 h. All experiments were carried out in duplicate. Three parallel cultures were used to determine concentrations of bacteria and transconjugants for all experiments. One culture served as a time (*t*) equals 0 culture, one culture served as a *t* = 2 h non-shaken culture, and the final culture served as a *t* = 2 h shaken culture (~200 rpm). After incubation at the designated temperature, 250 μl of each culture was plated onto LB agar plates (Fisher Scientific, Waltham, MA, USA) supplemented with TET (20 μg/ml) and CHL (20 μg/ml) and incubated for ~40 h at 37°C. A 1:100 dilution of each culture was read on an ATTUNE flow cytometer (Thermo-Fisher, Waltham, MA, USA) to obtain the concentrations of ER1793_mTag/pUUH239.2 and ER1793_SYFP2-Cat in each culture. Traditional plate counts were performed to verify flow cytometry bacterial concentrations. In instances where plate counts and flow cytometry numbers were not in good agreement, the higher of the two bacterial counts was used in order to produce a more conservative conjugation frequency. This situation occurred only once in an experiment involving AMP at 25 μg/ml. In this experiment, the concentrations determined by the flow cytometer were ~40% of the concentrations determined by plate counts. Potential transconjugants that grew on the LB agar plates supplemented with TET and CHL were confirmed by restreaking onto separate LB agar plates supplemented with AMP (100 μg/ml), KAN (50 μg/ml), CFX (25 μg/ml), TET (20 μg/ml), CHL (20 μg/ml), TET (20 μg/ml) + CHL (20 μg/ml), and GEN (25 μg/ml). Successful transconjugants were able to grow on all plates except the LB agar plates supplemented with GEN. Any potential transconjugant that failed to grow on all plates except GEN was regarded as not being a transconjugant.

Conjugation frequency (*Fc*) was determined by dividing the average number of transconjugants by the total number of bacteria:

(1) $$\begin{array}{l} F_{c}=\frac{C_{TC}}{C_{D}+C_{R}+C_{TC}} \end{array}$$

where *C*_*TC*_ (N L^−3^; N and L denotes the number of microbes and unit of length, respectively) is the concentration (number per volume) of transconjugants, *C*_*D*_ (N L^−3^) is the concentration of donor bacteria (ER1793 mTag/pUUH239.2), and *C*_*R*_ (N L^−3^) is the concentration of recipient bacteria (ER1793 SYFP2-Cat). The formula used to calculate conjugation frequency varies in the literature (e.g., Lopatkin et al., 2016 vs. Moller et al., 2017 vs. Jutkina et al., 2018). We chose a more conservative formula that was based on the total population of the bacterial culture, while many papers use a formula which only takes into account recipient bacteria (Muela et al., 1994; Zhang et al., 2013; Matsumoto et al., 2016; Moller et al., 2017; Jutkina et al., 2018). Conjugation requires both a donor and a recipient and omitting the donor (or recipient) population, particularly if the initial inoculate contains more donor or recipient bacteria, artificially inflates conjugation frequencies.

Similar to other authors (e.g., Schuurmans et al., 2014; Lopatkin et al., 2016), we found a high level of variability in *Fc* between experiments, particularly among our non-antibiotic controls. Standard deviations for *Fc* are presented in Table 1. In theory, the non-antibiotic control cultures used in each experiment (i.e., LB Broth, 1 mM NaCl, etc.) are identical and should yield a similar number of transconjugants in each experiment, but in reality this was not the case. This variability between experiments has been attributed by other investigators to differences in bacterial growth among different cultures (Lopatkin et al., 2016), and we strongly suspect this to be the case in our experiments. Despite our best efforts to attempt to control these factors, we were not always successful. As a result, we decided to pool the data for cultures with the same solution chemistries from experiments conducted on different days, particularly the non-antibiotic control cultures. The downside is that this generated very large standard deviations and resulted in less statistical significance in the data, but it did allow for more consistent comparisons with cultures supplemented with antibiotics and in the end, the large scale trends were still statistically significant.

**Table 1:** Conjugation frequencies at various physicochemical parameters.

**Solution**	**Temperature (C)**	**[Antibiotic] (ug/ml)**	**Agitation**	**Conjugation Frequency**	**Standard Deviation**
LB	37	0	Non-Shaken	1.36E-06	1.13E-06
LB+CFX	37	0.1	Non-Shaken	8.29E-07	2.26E-07
LB+CFX	37	25	Non-Shaken	1.73E-07	7.02E-08
LB+AMP	37	1	Non-Shaken	8.65E-07	2.39E-07
LB+AMP	37	25	Non-Shaken	2.67E-06	7.87E-07
LB+AMP	37	100	Non-Shaken	4.04E-07	1.56E-07
LB+KAN	37	1	Non-Shaken	6.53E-07	2.40E-07
LB+KAN	37	50	Non-Shaken	0.00E+00	0.00E+00
LB+GEN	37	1	Non-Shaken	1.95E-07	1.26E-07
LB+GEN	37	25	Non-Shaken	0.00E+00	0.00E+00
LB	37	0	Shaken	5.70E-06	4.64E-06
LB+CFX	37	0.1	Shaken	4.85E-06	1.34E-06
LB+CFX	37	25	Shaken	1.83E-06	4.03E-07
LB+AMP	37	1	Shaken	5.40E-06	1.45E-06
LB+AMP	37	25	Shaken	6.55E-06	1.40E-06
LB+AMP	37	100	Shaken	1.31E-06	2.18E-07
LB+KAN	37	1	Shaken	4.80E-06	1.53E-06
LB+KAN	37	50	Shaken	0.00E+00	0.00E+00
LB+GEN	37	1	Shaken	1.64E-06	5.63E-07
LB+GEN	37	25	Shaken	0.00E+00	0.00E+00
LB	22	0	Non-Shaken	1.63E-07	1.30E-07
LB+CFX	22	25	Non-Shaken	6.53E-08	1.21E-07
LB	22	0	Shaken	2.79E-07	1.43E-07
LB+CFX	22	25	Shaken	9.06E-08	1.28E-07
LB	4	0	Non-Shaken	0.00E+00	0.00E+00
LB+CFX	4	25	Non-Shaken	0.00E+00	0.00E+00
LB	4	0	Shaken	0.00E+00	0.00E+00
LB+CFX	4	25	Shaken	0.00E+00	0.00E+00
1 mM NaCl	37	0	Non-Shaken	1.92E-07	2.90E-07
1 mM NaCl+CFX	37	0.1	Non-Shaken	1.28E-07	2.03E-07
1 mM NaCl+CFX	37	25	Non-Shaken	1.47E-07	1.56E-07
1 mM NaCl+AMP	37	1	Non-Shaken	2.17E-07	2.24E-07
1mM NaCl+AMP	37	25	Non-Shaken	2.29E-07	1.65E-07
1 mM NaCl+AMP	37	100	Non-Shaken	9.06E-07	5.87E-07
1 mM NaCl+KAN	37	1	Non-Shaken	0.00E+00	0.00E+00
1 mM NaCl+KAN	37	50	Non-Shaken	0.00E+00	0.00E+00
1 mM NaCl+GEN	37	1	Non-Shaken	0.00E+00	0.00E+00
1 mM NaCl+GEN	37	25	Non-Shaken	0.00E+00	0.00E+00
1mM NaCl	37	0	Shaken	6.05E-07	9.12E-07
1mM NaCl+CFX	37	0.1	Shaken	4.21E-07	2.11E-07
1 mM NaCl+CFX	37	25	Shaken	9.68E-08	1.67E-07
1 mM NaCl+AMP	37	1	Shaken	5.67E-07	2.84E-07
1 mM NaCl+AMP	37	25	Shaken	1.94E-06	5.13E-07
1 mM NaCl+AMP	37	100	Shaken	1.24E-06	4.33E-07
1 mM NaCl+KAN	37	1	Shaken	0.00E+00	0.00E+00
1 mM NaCl+KAN	37	50	Shaken	0.00E+00	0.00E+00
1 mM NaCl+GEN	37	1	Shaken	0.00E+00	0.00E+00
1 mM NaCl+GEN	37	25	Shaken	0.00E+00	0.00E+00
1 mM NaCl	22	0	Non-Shaken	0.00E+00	0.00E+00
1 mM NaCl+CFX	22	25	Non-Shaken	0.00E+00	0.00E+00
1 mM NaCl	22	0	Shaken	0.00E+00	0.00E+00
1 mM NaCl+CFX	22	25	Shaken	0.00E+00	0.00E+00
1 mM NaCl	4	0	Non-Shaken	0.00E+00	0.00E+00
1 mM NaCl+CFX	4	25	Non-Shaken	0.00E+00	0.00E+00
1 mM NaCl	4	0	Shaken	0.00E+00	0.00E+00
1 mM NaCl+CFX	4	25	Shaken	0.00E+00	0.00E+00
10 mM NaCl	37	0	Non-Shaken	1.40E-06	1.20E-06
10 mM NaCl+CFX	37	0.1	Non-Shaken	2.30E-07	2.30E-07
10 mM NaCl+CFX	37	25	Non-Shaken	1.24E-05	4.56E-06
10 mM NaCl+AMP	37	1	Non-Shaken	9.45E-08	1.22E-07
10 mM NaCl+AMP	37	25	Non-Shaken	5.10E-07	2.67E-07
10 mM NaCl+AMP	37	100	Non-Shaken	1.54E-07	1.54E-07
10 mM NaCl+KAN	37	1	Non-Shaken	0.00E+00	0.00E+00
10 mM NaCl+KAN	37	50	Non-Shaken	0.00E+00	0.00E+00
10 mM NaCl+GEN	37	1	Non-Shaken	0.00E+00	0.00E+00
10 mM NaCl+GEN	37	25	Non-Shaken	0.00E+00	0.00E+00
10 mM NaCl	37	0	Shaken	6.02E-06	5.02E-06
10 mM NaCl+CFX	37	0.1	Shaken	1.90E-06	4.42E-07
10 mM NaCl+CFX	37	25	Shaken	9.70E-06	4.45E-06
10 mM NaCl+AMP	37	1	Shaken	1.68E-06	7.23E-07
10 mM NaCl+AMP	37	25	Shaken	2.57E-06	6.64E-07
10 mM NaCl+AMP	37	100	Shaken	3.76E-07	3.61E-07
10mM NaCl+KAN	37	1	Shaken	0.00E+00	0.00E+00
10 mM NaCl+KAN	37	50	Shaken	0.00E+00	0.00E+00
10 mM NaCl+GEN	37	1	Shaken	0.00E+00	0.00E+00
10 mM NaCl+GEN	37	25	Shaken	0.00E+00	0.00E+00
10 mM NaCl	22	0	Non-Shaken	0.00E+00	0.00E+00
10 mM NaCl+CFX	22	25	Non-Shaken	0.00E+00	0.00E+00
10 mM NaCl	22	0	Shaken	4.50E-08	1.19E-07
10 mM NaCl+CFX	22	25	Shaken	0.00E+00	0.00E+00
10 mM NaCl	4	0	Non-Shaken	0.00E+00	0.00E+00
10 mM NaCl+CFX	4	25	Non-Shaken	0.00E+00	0.00E+00
10 mM NaCl	4	0	Shaken	0.00E+00	0.00E+00
10 mM NaCl+CFX	4	25	Shaken	0.00E+00	0.00E+00
100 mM NaCl	37	0	Non-Shaken	7.20E-08	1.69E-07
100 mM NaCl+CFX	37	0.1	Non-Shaken	7.68E-08	9.94E-08
100 mM NaCl+CFX	37	25	Non-Shaken	3.76E-07	2.79E-07
100 mM NaCl+AMP	37	1	Non-Shaken	1.23E-07	1.36E-07
100mM NaCl+AMP	37	25	Non-Shaken	2.08E-08	5.50E-08
100 mM NaCl+AMP	37	100	Non-Shaken	0.00E+00	0.00E+00
100mM NaCl+KAN	37	1	Non-Shaken	0.00E+00	0.00E+00
100 mM NaCl+KAN	37	50	Non-Shaken	0.00E+00	0.00E+00
100 mM NaCl+GEN	37	1	Non-Shaken	0.00E+00	0.00E+00
100 mM NaCl+GEN	37	25	Non-Shaken	0.00E+00	0.00E+00
100 mM NaCl	37	0	Shaken	2.90E-07	3.24E-07
100 mM NaCl+CFX	37	0.1	Shaken	4.82E-07	3.43E-07
100 mM NaCl+CFX	37	25	Shaken	1.28E-06	5.09E-07
100 mM NaCl+AMP	37	1	Shaken	3.44E-07	1.72E-07
100 mM NaCl+AMP	37	25	Shaken	1.46E-07	1.55E-07
100 mM NaCl+AMP	37	100	Shaken	0.00E+00	0.00E+00
100 mM NaCl+KAN	37	1	Shaken	0.00E+00	0.00E+00
100 mM NaCl+KAN	37	50	Shaken	0.00E+00	0.00E+00
100mM NaCl+GEN	37	1	Shaken	0.00E+00	0.00E+00
100 mM NaCl+GEN	37	25	Shaken	0.00E+00	0.00E+00
100 mM NaCl	22	0	Non-Shaken	0.00E+00	0.00E+00
100 mM NaCl+CFX	22	25	Non-Shaken	0.00E+00	0.00E+00
100 mM NaCl	22	0	Shaken	0.00E+00	0.00E+00
100 mM NaCl+CFX	22	25	Shaken	0.00E+00	0.00E+00
100 mM NaCl	4	0	Non-Shaken	0.00E+00	0.00E+00
100 mM NaCl+CFX	4	25	Non-Shaken	0.00E+00	0.00E+00
100mM NaCl	4	0	Shaken	0.00E+00	0.00E+00
100 mM NaCl+CFX	4	25	Shaken	0.00E+00	0.00E+00

### Statistics

Two sample *T*-test assuming unequal variances (two-tailed) were carried out using Microsoft Excel 2010 (Microsoft, Redmond, WA, USA) and corrected for multiple comparisons using the Bonferroni correction. *p* < 0.003 were considered significant.

## Results

### Shaken vs. non-shaken cultures

**Figure 1:**
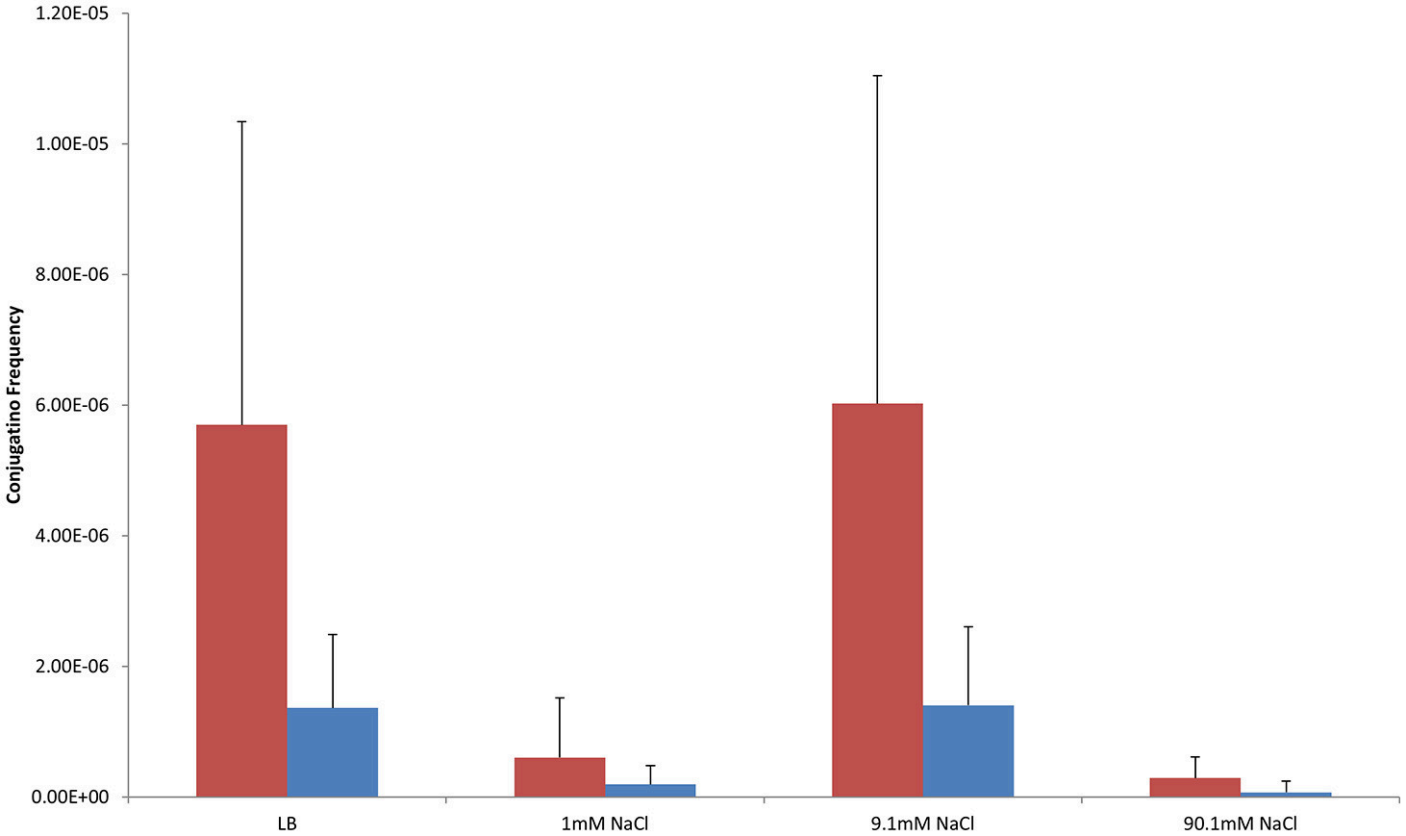
Conjugation frequencies in shaken and non-shaken cultures at 37°C without antibiotics. Red-Shaken Cultures, Blue-Non-shaken cultures. Error bars represent standard deviation.

**Figure 2:**
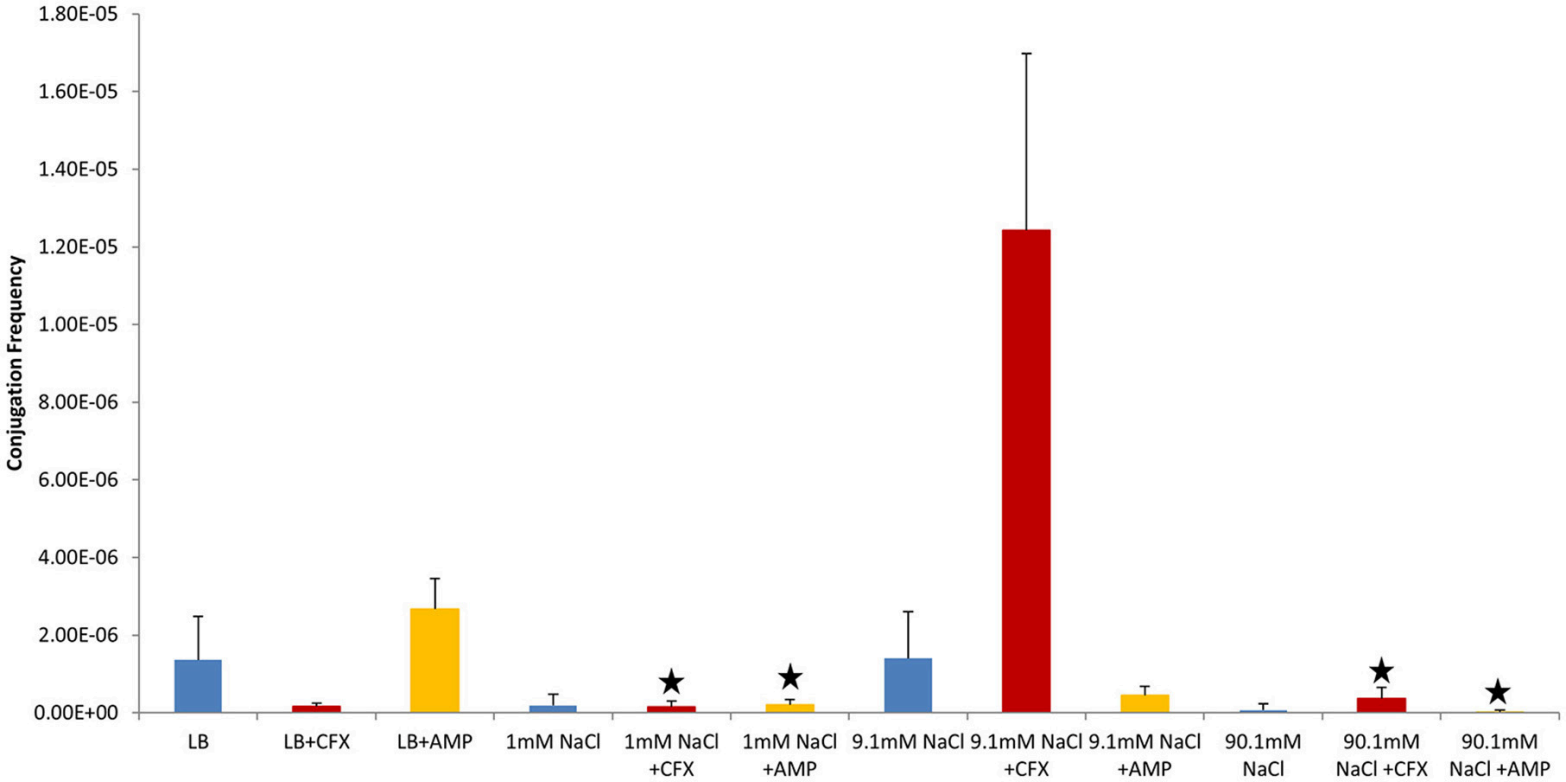
Conjugation frequencies at 37°C in the presence of antibiotics, Non-shaken cultures. Blue control cultures (no antibiotics). Red-CFX(25 μg/ml), Orange-AMP(25 μg/ml). Black star = not statistically significant relative to control culture of same solution chemistry. See Materials and Methods section for description of antibiotic abbreviations. Error bars represent standard deviation.

**Figure 3:**
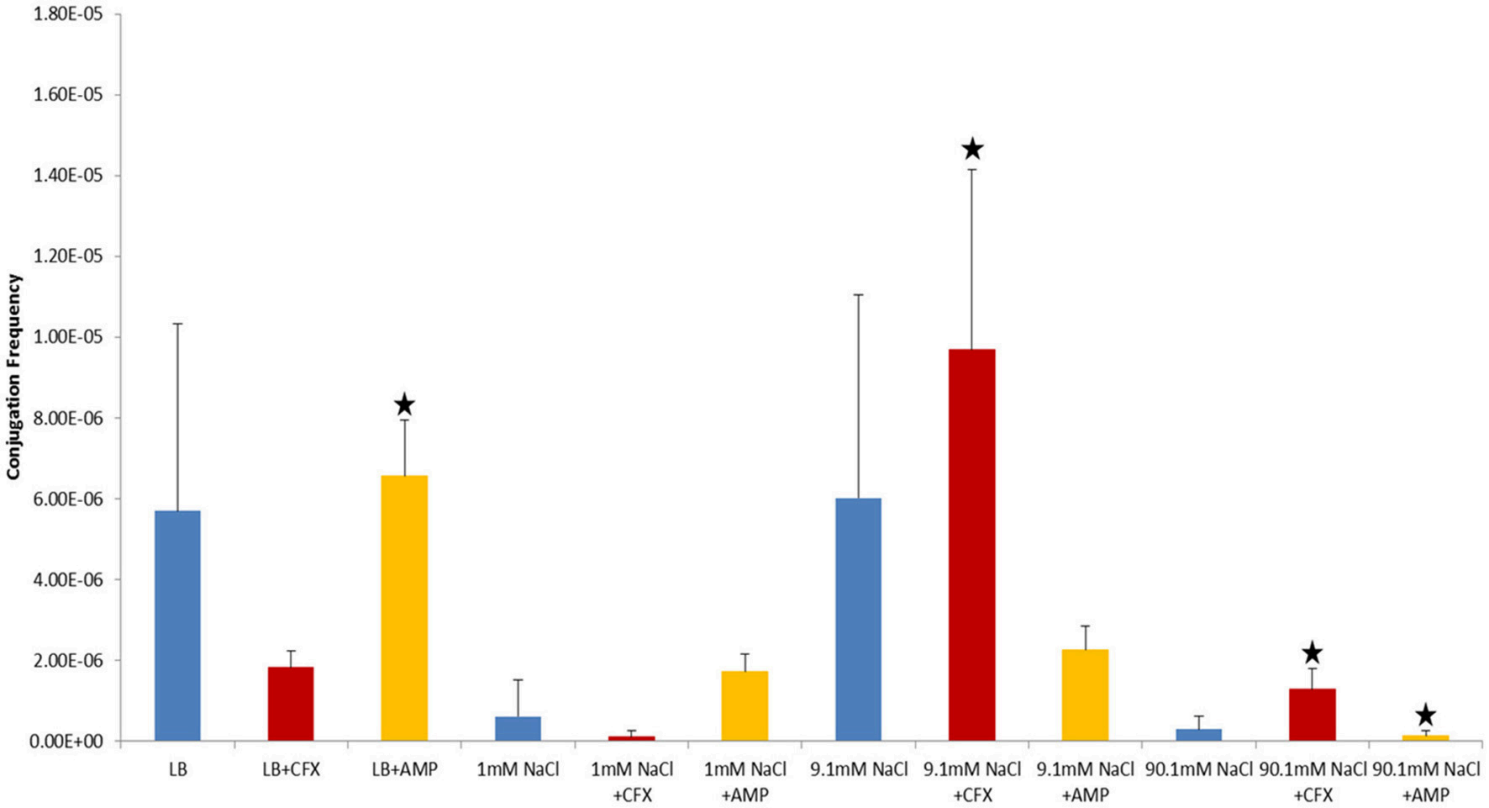
Conjugation frequencies at 37°C in the presence of antibiotics, Shaken cultures. Blue control cultures (no antibiotics). Red-CFX(25 μg/ml), Orange-AMP(25 μg/ml). Black star = not statistically significant relative to control culture of same solution chemistry. See Materials and Methods section for description of antibiotic abbreviations. Error bars represent standard deviation.

Shaken cultures had higher conjugation frequencies than non-shaken cultures in non-antibiotic cultures (Figure 1). The observation was also generally true for cultures supplemented with antibiotics with the notable exception of non-shaken cultures in 9.1 mM NaCl supplemented with 25 μg/ml of CFX which had much higher conjugation frequencies (Table 1, Figures 2, 3).

### Temperature

Conjugation frequency increased with temperature. Most conjugation occurred at 37°C. There was no conjugation in any cultures at 4°C and very little conjugation at 22°C (Table 1). At 22°C, conjugation only occurred in LB cultures without antibiotics, LB supplemented with 25 μg/ml of CFX, and in the 9.1 mM NaCl shaken cultures without antibiotics (Table 1).

### Salinity

In control cultures (not supplemented with antibiotics), the conjugation frequency in the growing LB culture exceeded all non-growing cultures (both shaken and non-shaken cultures) except 9.1 mM NaCl. The non-growing culture in 9.1 mM NaCl had a similar conjugation frequency in both shaken and non-shaken cultures (*p* = 0.398, *p* = 0.3419 respectively) (Figure 1). The optimal salinity for conjugation in non-growing cultures was found to be 9.1 mM NaCl, especially when supplemented with CFX at 25 μg/ml in both shaken and non-shaken cultures (Figures 2,3).

### Antibiotic type and concentration

**Figure 4:**
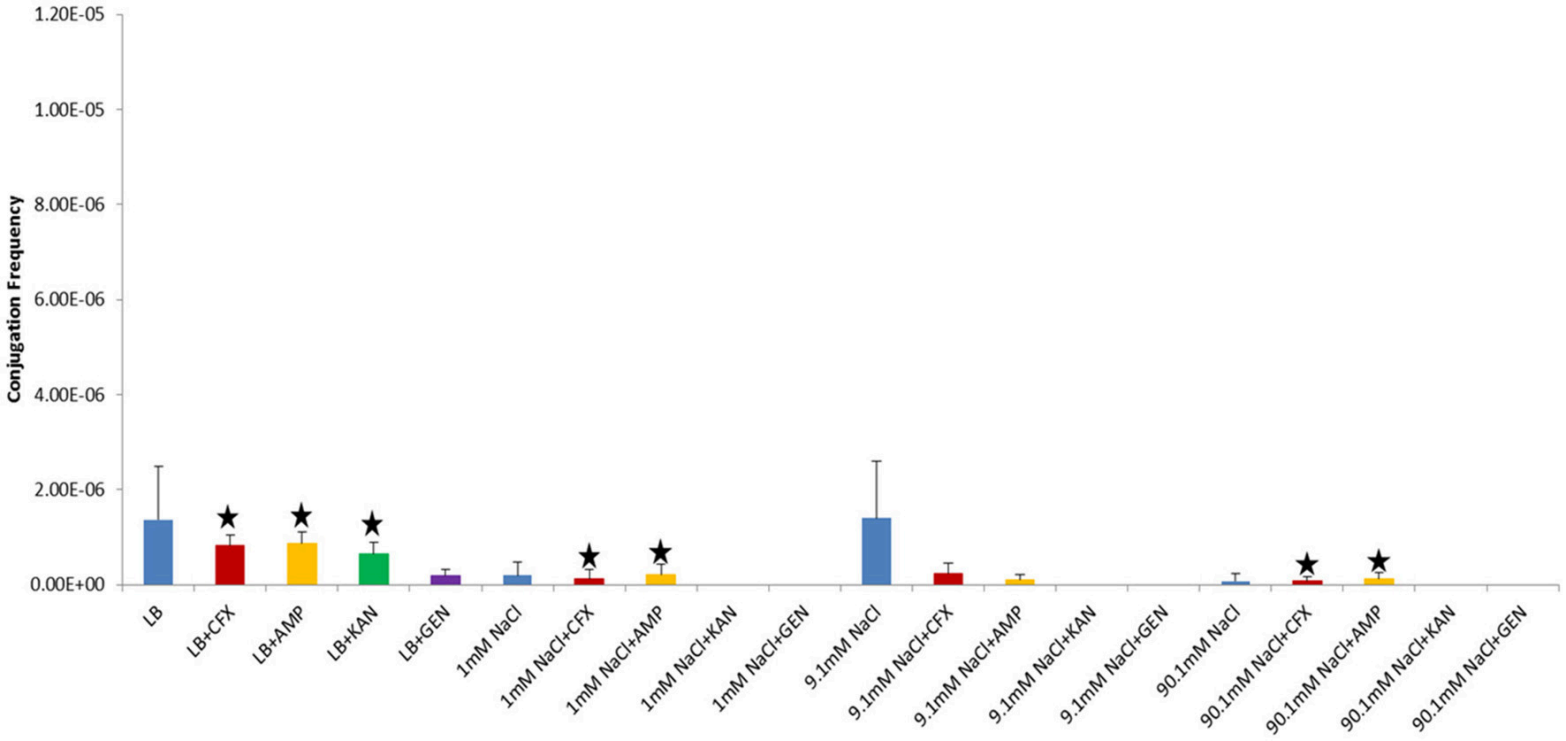
Conjugation frequencies in the presence of sub-inhibitory concentrations of antibiotics at 37°C, Non-shaken cultures. Blue-Control cultures (no antibiotics), Red-CFX(0.1 μg/ml), Orange-AMP(1 μg/ml), Green-KAN(1 μg/ml), Purple-GEN(1 μg/ml). Black star = not statistically significant relative to control culture of same solution chemistry. See Materials and Methods section for description of antibiotic abbreviations. Error bars represent standard deviation.

**Figure 5:**
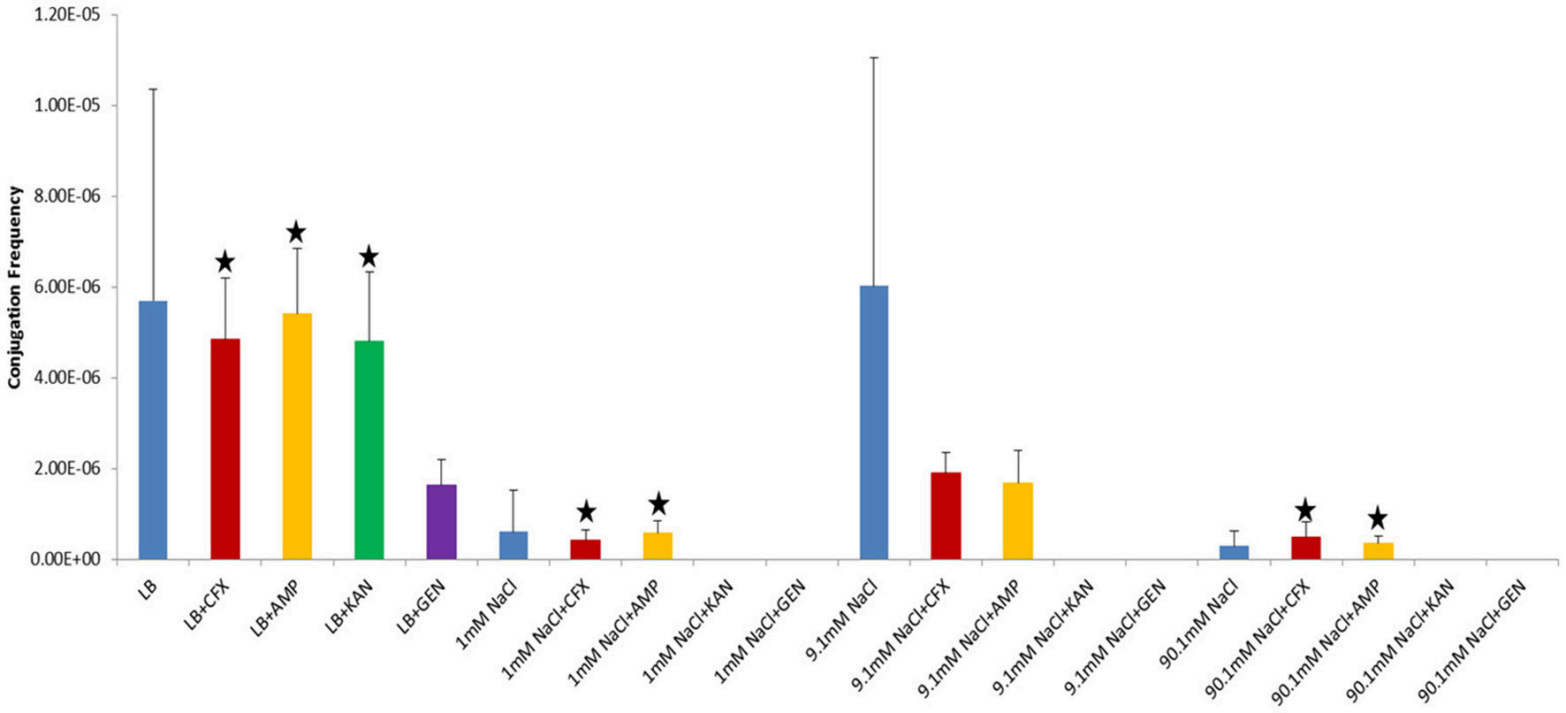
Conjugation frequencies in the presence of sub-inhibitory concentrations of antibiotics at 37°C, Shaken cultures. Blue-Control cultures (no antibiotics), Red-CFX(0.1 μg/ml), Orange-AMP(1 μg/ml), Green-KAN(1 μg/ml), Purple-GEN(1 μg/ml). Black star = not statistically significant relative to control culture of same solution chemistry. See Materials and Methods section for description of antibiotic abbreviations. Error bars represent standard deviation.

In our study, donor and recipient bacteria did not possess resistance to GEN and only donors and transconjugants possessed resistance to AMP, CFX, and KAN. No conjugation was observed in cultures supplemented with GEN (25 μg/ml) or KAN (50 μg/ml). At subinhibitory concentrations of GEN (1 μg/ml), conjugation occurred in LB at a lower frequency than the control culture in both shaken and non-shaken cultures (*p* = <0.0001) (Figures 4, 5, Table 1). At subinhibitory concentrations of KAN (1 μg/ml), the conjugation frequency in LB was similar to the control in both non-shaken cultures (*p* = 0.003) and shaken cultures (*p* = 0.748). At subinhibitory concentrations of CFX (0.1 μg/ml) and AMP (1 μg/ml), the conjugation frequencies of the CFX and AMP supplemented cultures were similar to one another in all solutions in both shaken and non-shaken cultures (Figures 4, 5, Table 1.). At high concentrations of AMP (100 μg/ml), the conjugation frequency was generally less than cultures containing 25 μg/ml AMP or similar (i.e. not statistically significant) (Table 1).

Conjugation in the presence of CFX and AMP at 25 μg/ml at 37°C generally produced opposing results (i.e., when conjugation frequencies in CFX supplemented cultures were high, AMP supplemented cultures were low and vice-versa in both shaken and non-shaken cultures) (Figures 2, 3). In growing cultures, the conjugation frequency in the presence of AMP was nearly double that of controls in non-shaken cultures (*p* = 0.0023) and similar to that of the control in shaken cultures (*p* = 0.339), while in CFX supplemented cultures, the conjugation frequency was lower than the control (*p* = <0.0001) (Figures 2, 3, Table 1). However, in 9.1 mM NaCl the conjugation frequency in the presence of CFX (25 μg/ml) was 8.8 times higher than the 9.1 mM NaCl control (*p* = <0.0001) and 9.1 times higher than the conjugation frequency of the LB control (*p* = <0.0001) (Figure 2). In shaken cultures, the trend was similar, but, not statistically significant (the conjugation frequency was 1.6 times higher than the 9.1 mM NaCl control (*p* = 0.0189) and 1.7 times higher than the LB culture (*p* = 0.0106) (Figure 3, Table 1). Cultures supplemented with AMP at 25 μg/ml were lower than the non-antibiotic control in 9.1 mM NaCl in both non-shaken (*p* = <0.0001) and shaken (*p* = <0.0001) cultures. The conjugation frequency was 3.2 times higher than the control (*p* = <0.0001) in 1 mM NaCl shaken cultures supplemented with 25 μg/ml AMP. Shaken CFX supplemented cultures in 90.1 mM NaCl had higher conjugation frequencies (4.4 times higher) than the controls, but were not statistically significant (*p* = 0.04) (Figure 3, Table 1).

## Discussion

The increasing prevalence of ARG in pathogenic bacteria necessitates that the mechanisms and conditions in which antibiotic resistance is spread via HGT in bacteria be studied in more detail. While there has been much written in the literature in regards to the presence of antibiotics in the environment increasing rates of HGT, few studies have examined non-growing cultures. We examined the frequency of conjugation in both growing and non-growing bacteria cultures and found that in certain solution chemistries, most notably 9.1 mM NaCl, the frequency of conjugation in non-growing *E. coli* exceeded that of growing cultures, especially in the presence of CFX at 25 μg/ml. While the results from our experiments are to a large degree a reflection of the plasmid and bacterial strain we utilized, the general trends we observed related to solution chemistry and β-lactam antibiotics likely hold true for other plasmid/bacteria systems as well. However, additional research is warranted to confirm that these observations also apply for other non-growing bacteria and environmental conditions, The experimental setup we utilized was an optimal scenario for successful conjugation of pUUH239.2 and *E. coli* ER1793 since the bacterial strain is restriction enzyme deficient. It should be noted that the conjugation frequency for pUUH239.2 in *Klebsiella pneumoniae* (the species from which the plasmid was originally isolated) has been reported to be much higher than that of *E. coli* (Sandegren et al., 2012). Though we have not tested, we would expect that the trends reported in this paper to be similar to that in *Klebsiella pneumoniae* and any other bacteria that the plasmid can be transmitted to via conjugation.

### Optimal physiochemical conditions

In general, we found that physiochemical parameters that favored growth enhanced conjugation in both growing and non-growing cultures. We found that conjugation occurred most frequently in cultures that were shaken at 37°C. This temperature is likely reflective of the optimal growth temperature for *E. coli* (and many other human pathogens). Other studies have also reported varying degrees of increased conjugation with increasing temperature (Khalil and Gealt, 1987; Fernandez-Astorga et al., 1992). Other studies have found increased conjugation in growing bacteria in the exponential phase (Muela et al., 1994; Händel et al., 2015; Lopatkin et al., 2016). It is important to note that we harvested our bacteria during exponential phase prior to re-suspending them in NaCl solutions. The average time from the beginning of harvesting to inoculation of cultures at *t* = 0 was 30–45 min during which time the bacteria were primarily suspended in 1 mM NaCl. Presumably, the bacteria shifted from exponential to stationary phase during this time, but it is unclear how long it takes bacteria to make the transition or to what extent this may have impacted the conjugation results in the non-growing cultures.

Bacterial cultures are commonly shaken to ensure proper aeration, nutrient availability, and to prevent aggregation and/or biofilm formation. Our results clearly demonstrate that shaking at ~200 rpm does not prevent donor and recipient cells from contacting one another or subsequent conjugation from occurring. Increased aeration via shaking cultures generally leads to more growth in *E. coli* cultures and other studies have reported conjugation in shaken cultures at 200 rpm (Händel et al., 2015). In addition to aeration, shaken cultures can have improved access to nutrients and removal of waste which can lead to higher bacterial concentrations, even in the absence of oxygenation (Juergensmeyer et al., 2007). In our study, there was generally more conjugation in shaken cultures than non-shaken cultures (the non-shaken 9.1 mM culture supplemented with CFX had a higher conjugation frequency relative to its shaken counterpart, but it was not statistically significant (*p* = 0.0276)). Presumably, in non-growing cultures the primary advantage of shaking could be oxygenation, but we were unable to obtain oxygen concentrations for our shaken and non-shaken cultures to verify this assumption and/or conclude that increased conjugation in shaken cultures was due to increased oxygenation. We are not aware of a study examining an association of shaking (or any other variable) with increased conjugation in non-growing bacterial cultures.

### Influence of salinity on conjugation

This study was the first to our knowledge to systematically investigate the influence of solution salinity (1, 9.1, and 90 mM NaCl) on conjugation in non-growing cultures. We chose to conduct our experiments over this range of NaCl concentrations because these salinities spanned the range of unfavorable (low ionic strength) and favorable (high ionic strength) interaction conditions for bacteria that are typically employed in studies examining their fate in the environment (Walker et al., 2004; Torkzaban et al., 2008; Kim et al., 2009). This observed influence of solution salinity on conjugation under non-growing conditions is expected to be related to differences in the strength of cell-cell interactions and changes in the conformation of cell surface structures. In particular, interaction energy calculations for bacteria demonstrate that increasing the ionic strength of monovalent electrolyte solutions tends to diminish the energy barrier to cell-cell interaction due to a reduction in the magnitude of the cell surface charge and compression of the double layer thickness (Rijnaarts et al., 1999). In addition, the cell surface structures impart nanoscale roughness features that have a large influence on the energy barrier height and the strength of cell-cell interactions (Bradford et al., 2017). The morphology of surface proteins and cell roughness is expected to change with the solution ionic strength. In particular, a more open conformation of surface proteins is expected at a lower ionic strength and in the absence of divalent cations (Kim et al., 2009). As a result, it might be more difficult for the pilus to extend freely in a 90 mM NaCl solution. In lower salinity solutions such as 1 mM NaCl, the pilus would be free to extend into solution, but the large energy barrier would be expected to keep bacteria further apart from one another, possibly impeding conjugation. In a moderate salinity solution, such as 9.1 mM NaCl, the energy barrier would be less, allowing bacteria to come closer to one another and the pilus would be free to extend into the solution and conjugation would be more likely.

A few authors have reported increased conjugation with increasing salinity in the presence of growth media (Singleton, 1983, 1984; Hayashi et al., 1988; Beuls et al., 2012). However, all of these studies examined conjugation at much higher salinities than we examined. Singleton (1983) reported 2.3 times more transconjugants in 100 mM NaCl than in his control cultures of 50 mM phosphate buffer. However, Singleton diluted the donor-recipient mixture in nutrient broth after 5 min of mating in phosphate buffer and then let the culture sit for another 30 min. This would have raised the salinity considerably (possibly by 85 mM NaCl, depending on the exact formulation of the nutrient broth used) and still allowed the bacteria to grow and/or conjugate. The LB broth used in our experiments is approximately 171 mM NaCl, by comparison Singleton's 100 mM culture may have been actually closer to 200 mM NaCl. While Singleton's work does show increased conjugation with increased salinity, his experiments were more similar to our experiments conducted in LB than in non-growing cultures. In LB, the effects of the high salinity could be masked by the presence of organic matter in the form of yeast extract which could alter surface charges and interaction energies allowing for more conjugation to occur than in pure NaCl solutions. Neither Singleton (1983) nor Hayashi et al. (1988) conducted experiments between 0 and 80 mM NaCl, however both reported declines in conjugation frequency above approximately 200 mM NaCl, suggesting an optimal salinity for conjugation. Beuls et al. (2012) attributed increased conjugation at higher salinities (~855 mM NaCl) to *B. thuringiensis* forming chains and thus having a more stable contact with neighboring cells. However, our *E. coli* did not form chains at the examined salinities.

### Influence of antibiotics on conjugation

We tested different antibiotics to see if conjugation in the presence of antibiotics was a general response or specific to the antibiotic used. We found no conjugation in the presence of selective concentrations of the aminoglycosides GEN or KAN (25 and 50 μg/ml, respectively) and similar or lower conjugation frequencies to controls in LB cultures at subinhibitory concentrations (GEN and KAN at 1 μg/ml). These data suggests that exposure to antibiotics in general will not induce HGT via conjugation. In the plasmid/bacteria system we utilized it tended to decrease conjugation frequencies. Only in the presence of β-lactam antibiotics did conjugation increase. As noted above, conjugation in the presence of CFX and AMP yielded opposing results. The presence of AMP at 25 μg/ml increased conjugation frequencies relative to the non-antibiotic controls in LB (non-shaken cultures) and 1 mM NaCl (shaken) cultures, while the presence of CFX at 25 μg/ml increased conjugation frequencies in cultures of 9.1 mM non-shaken cultures) and 90.1 mM NaCl (shaken cultures). Higher and lower (subinhibitory) concentrations did not result in increased conjugation. Other studies have reported less conjugation at high concentration of antibiotics (Licht et al., 2003; Händel et al., 2015) and at subinhibitory concentrations as well (Lopatkin et al., 2016). Our results suggest that specific antibiotics, in this case β-lactams, can promote HGT via conjugation under certain conditions.

β-lactam antibiotics inhibit cell wall synthesis by binding to penicillin binding proteins (PBPs) which are transpeptidases responsible for cross-linking peptidoglycan fragments in the cell wall (Zeng and Lin, 2013). As a result, β-lactams are only effective against growing bacteria. AMP and CFX selectively bind to different PBPs. AMP targets the PBPs for peptidoglycan hydrolysis: PBPs 4, 7, and 8 (PBP8 is a degradation product of PBP7) (Henderson et al., 1994; Kocaoglu and Carlson, 2015), while CFX is selective for PBP 3 (Kocaoglu and Carlson, 2015), which functions in peptidoglycan synthesis. In growing cells, PBP3 is one of about a dozen proteins that form the septal ring, which is responsible for forming the septum in dividing bacteria (Weiss et al., 1999). PBP3 only binds to the septal ring after the ring forms and after at least three other proteins have bound to the ring (Weiss et al., 1999; Mercer and Weiss, 2002; Piette et al., 2004). When CFX binds to PBP3 the septum fails to form properly and cells continue to elongate into a filamentous morphology rather than divide. It does this regardless of whether the cell possesses resistance to β-lactams or not (Kjeldsen et al., 2015b). Visual analysis of donor (ER1793_mTag/pUUH239.2) and recipient (ER1793_SYFP2-cat) bacteria showed that growing cultures supplemented with 25 μg/ml CFX took on a filamentous morphology, while non-growing cells did not. This observation is consistent with CFX preferentially binding to PBP3 in growing cultures. By contrast, PBPs 4, 7, and 8 are reported to be loosely associated with the cytoplasmic membrane (Sauvage et al., 2008). Visual analysis of donor and recipient bacteria showed that growing cultures supplemented with 25 μg/ml AMP did not take on a filamentous morphology (nor did any of the non-growing cultures, regardless of exposure to CFX or AMP) and were visually indistinguishable from cells not supplemented with antibiotics. LB cultures supplemented with CFX (25 μg/ml) formed less transconjugants than the LB controls or cultures supplemented with AMP (25 μg/ml) in both non-shaken and shaken cultures (as opposed to having no effect like the shaken AMP culture). It is intriguing to suggest that the morphological differences associated with growing cultures in CFX compared to AMP could have impacted the ability of CFX exposed cells to conjugate. But we can provide no evidence for this assertion at this time other than the observation that control LB cultures and LB+AMP cultures with rod shaped bacteria (as opposed to filamentous shape in CFX) had higher conjugation frequencies (Figures 2, 3).

In order for β-lactams to reach their target PBPs, they typically most avoid being hydrolyzed by β-lactamases. Numerous studies have reported the induction of β-lactamases in growing cells by fragments of the cell wall, inactivation of PBPs, and antibiotics (Minami et al., 1980; Moya et al., 2009; Zeng and Lin, 2013; Kjeldsen et al., 2015b). In growing cells, we would expect some β-lactamases to be produced, either the chromosomal ampC and/or the pUUH239.2 encoded blaCTX-M-15. The promoter for the blaCTX-M-15 gene which codes for the production β-lactamase on pUUH239.2 is ISEcp1, which has been shown to be inducible by CFX, particularly in growing cells (Kjeldsen et al., 2015b). However, the induction of the plasmid encoded blaCTX-M-1 by CFX was found to produce lower amounts of β-lactamase when the gene was on a plasmid than when it was on the chromosome (Kjeldsen et al., 2015a). In growing cells we hypothesize that CFX binds to PBP3 and/or is hydrolyzed by β-lactamases to such an extent that CFX does not make it into the cytoplasm at high enough concentrations to induce conjugation. AMP also would be expected to be bind to its respective PBPs, but may not induce as much β-lactamase activity in *E. coli* as it is unclear whether AMP can induce blaCTX-M-15 on pUUH239.2 and chromosomal induction is unlikely given the lack of an *ampR* gene in *E. coli* (Honoré et al., 1986; Zeng and Lin, 2013). Thus, more AMP might make it to the cytoplasm where it could interact with chromosome or plasmid and induce conjugation in growing cells. In non-growing cells it is unclear whether β-lactamases would be produced at high enough levels to significantly hydrolyze AMP or CFX. We would expect that induction β-lactamases in non-growing cells to be less than that of growing cells due to reduced amounts of cell wall biosynthesis and a general reduction in the production of β-lactamases in non-growing *E. coli* relative to growing *E. coli* (Jaurin and Normark, 1979; Jaurin et al., 1981). This could allow both AMP and CFX to penetrate further into the cell in non-growing cultures than in growing cultures.

It is unclear what the fate of AMP and CFX is in non-growing cells. Studies examining the binding of β-lactams to PBPs tend to be conducted on non-growing cells (either extracted membranes or cells suspended in buffer), suggesting that β-lactams will bind to their respective PBPs if they are present. AMP has been reported to diffuse through the outer membrane via porins in *E. coli* at nearly double the rate of CFX (Nikaido et al., 1983; Yoshimura and Nikaido, 1985) and is more hydrophobic than CFX (Yoshimura and Nikaido, 1985), which might suggest that AMP would be more likely to penetrate membranes than CFX. But both (Kjeldsen et al., 2015a) and Moller et al. (2017) were able to induce expression of CTX-M-1 on a plasmid in growing bacteria with CFX, suggesting that CFX is capable of passing through the membranes and into the cytoplasm. In non-growing cells, we hypothesize that CFX does not bind to PBP3 in the plasma membrane because PBP3 is either not produced at all or produced at much lower levels in non-growing cells. Studies have found that bacteria in stationary phase produce much less PBPs than those in exponential phase (Mendelman and Chaffin, 1985; Stevens et al., 1993). We hypothesize that in non-growing cultures, CFX is making it into the cytoplasm due to a lack of PBP3 in the cell and decreased production of β-lactamases. It is unclear to us whether AMP enters the cytoplasm (and at what concentrations) or if much of it still binds to PBPs 4, 7, and 8 in the cell membranes.

Beaber et al. (2004) reported that the SOS response can promote HGT and Maiques et al. (2006) reported that β-lactam antibiotics could induce the SOS response in *S. aureus* leading to an increase in HGT. Recently, Moller et al. (2017) reported that Tra proteins (transfer proteins) on a plasmid containing CTX-M-1 were upregulated in the presence of high concentrations of CFX, but not low concentrations of CFX. The mechanism by which this occurs is unknown, but it did not make a difference whether the CTX-M-1 gene was on a plasmid or on the chromosome (Moller et al., 2017). This suggests that CFX in some way induces *tra* genes to increase conjugation. We hypothesize that in non-growing bacteria, CFX is able to diffuse into the cytoplasm to a greater extent than AMP and interact with the plasmid in such a way (possibly the blaCTX-M-15) to induce conjugation. It is unclear if AMP could induce the same reaction, but our results suggest it may not be as effective.

While we only examined a few variables associated with conjugation in this study, the results suggests that conjugation with pUUH239.2 only occurs over a relatively narrow range of environmental conditions, most notably temperature. While conjugation is relatively high in growing cultures, the presence of antibiotics did not generally increase conjugation frequency in growing cultures. In non-growing cells, the optimal salinity for conjugation was 9.1 mM NaCl. Thus, we would expect environments that could maintain a temperature of around 37°C and promote growth such as animal guts, compost heaps, manure piles and other nutrient rich environments to be ideal for pUUH239.2, especially in the absence of antibiotics. In non-growing cultures, salinity, and antibiotics become a factor in addition to temperature.

Environments such as thermal waters, some soils and other nutrient depleted environments that can reach a 37°C temperature could enhance transfer of pUUH239.2, especially in the presence of higher concentrations (~25 μg/ml) of antibiotics such as CFX. While our data suggests there is an optimal salinity range for conjugation in non-growing cultures with pUUH239.2, it is unclear how large the range is, but the mechanisms that we envision to govern this process would not be expected to be limited to pUUH239.2 and associated hosts. Further research is needed to constrain the range of conditions in which conjugation in non-growing bacteria can occur. Our results also suggests that HGT of ARG in non-growing cells might be as important a factor in the spread of ARG as in growing bacteria.

## Author contributions

BH and SB designed experiments, BH conducted experiments, BH and SB drafted the manuscript.

### Conflict of interest statement

The authors declare that the research was conducted in the absence of any commercial or financial relationships that could be construed as a potential conflict of interest.
